# The effects of DNA methylation and epigenetic factors on the expression of CD133 in ovarian cancers

**DOI:** 10.1186/1757-2215-5-28

**Published:** 2012-10-15

**Authors:** Kyung-Jin Min, Kyeong A So, Yung-Taek Ouh, Jin-Hwa Hong, Jae-Kwan Lee

**Affiliations:** 1 Department of Obstetrics and Gynecology, Guro Hospital, College of Medicine, Korea University, Seoul, Korea

**Keywords:** Cancer stem cell, CD133, Epigenetics, 5`-aza-2`-deoxycytidine, Trichostatin A

## Abstract

**Background:**

To identifying the effects of DNA methylation and epigenetic factors on the expression of CD133, a cancer stem cell marker, in gynecologic cancer cell lines.

**Methods:**

Ovarian cancer cell lines (OVCAR-8 and IGROV-1) and an endometrial cancer cell line (Ishikawa) were treated with 5-aza-2`-deoxycytidine (DAC) or Trichostatin A (TSA). Expression of CD133 was evaluated by quantitative real-time PCR, methylation-specific PCR (MSP), reverse transcription**-**PCR, western blot, and FACS analysis. All results are representative of three independent experiments.

**Results:**

CD133 mRNA expression varied among the different cell lines; the weakest expression was observed in OVCAR-8 cells, while it was strongly expressed in Ishikawa cells. The degree of methylation of the CD133 P2 promoter was 61% in OVCAR-8 cells, 53% in IGROV-1 cells, and 43% in Ishikawa cells. CD133 expression was increased at both the mRNA and protein level after DAC treatment. On the contrary, CD133 mRNA expression decreased after TSA treatment decreased in all cell lines except OVCAR-8. In addition, MSP of the CD133 P2 promoter revealed that methylation was reduced after treatment with either DAC or TSA.

**Conclusions:**

The expression of the CD133 antigen in primary ovarian and endometrial cancer cell lines is regulated by epigenetics, as indicated by its increased expression following DAC treatment and irregular expression pattern followed by TSA treatment. In addition, the expression of CD133 was negatively correlated with the degree of methylation of the CD133 P2 promoter.

## Background

Ovarian cancer is one of the most fatal gynecological cancers. The 2008 National Cancer Institute (NCI) statistics indicate that each year more than 20,000 new cases of ovarian cancer are diagnosed, with 15,000 deaths [1]. Although early detection of ovarian cancer is reported to increase the five-year survival rate by up to 92%, the rate of actual early detection is 20% or less, lowering the overall five-year survival rate to between 15% and 45% [1].

Chemotherapy is administered to ovarian cancer patients after surgery, as surgical treatment does not confer a sufficient treatment effect. Chemotherapy, however, is not very effective either; ovarian cancer recurs in the majority of advanced-case patients, and tolerance to chemotherapy may develop. Radiation therapy, immunotherapy, and hormonal therapy are also used as treatment methods, although their relative effectiveness has not been clearly demonstrated [2].

Interest in the relationship of cancer stem cells and their role in the response to treatment of ovarian cancer is on the rise. Cancer stem cells have specific genetic variations that give them the capability to limitlessly divide and proliferate, like other stem cells, in addition to the continuous production of various cancer cells. As a result of these capabilities, cancer stem cells can mediate cancer occurrence, tolerance to treatment, and consequently, recurrence [3].

The results of studies on the epigenetic mechanism involved in this process have been reported. Specifically, expression of CD133 in patients with hepatic cancer is associated with a poor prognosis. In addition, the expression of CD133 is significantly affected by gene methylation. It is known that TGF-β acts as an up-regulator of CD133 by promoter methylation of CD133 [4]. In addition, it is known that in cervical cancer, inactivation of the FHIT gene by 5`-CpG island methylation plays an important role in the occurrence of cervical cancer [5]. In endometrial cancer, CD133 expression may be epigenetically regulated and that cell fractions enriched for CD133+ cells may well contribute to endometrial cancer tumorigenicity, pathology and recurrence [6-8]. Among these studies, Friel et al. conducted the experiment with Ishikawa cell line as control.

Ovarian cancer stem cells, through selective carriers such as ABCG2 and MDR1, are known to be a major cause of tolerance to chemotherapy, metastasis, and recurrence of ovarian cancer [9,10]. As targeted treatment for ovarian cancer stem cells, which are resistant to treatment, is considered a more effective treatment modality for ovarian cancer, the identification of ovarian-cancer stem cells and a treatment that targets such cells is considered an effective strategy for the successful treatment of ovarian cancer [11]. In addition, the treatment of ovarian cancer stem cells could be advanced if their relationship with expression of the CD133 stem cell maker can be clarified by identifying promoter methylation.

Thus, in this study, the CD133 marker of ovarian cancer stem cells was examined, and the relationship between gene expression and promoter methylation were identified. It was compared CD133 expression in ovarian cancer cell lines and Ishikawa cell line as control.

## Methods

### Cell culture and cell treatments

Ovarian cancer cell lines (OVCAR-8 and IGROV-1) were cultured in RPMI 1640 supplemented with 10% fetal bovine serum (FBS), 500 units/ml penicillin and 500 μg/ml streptomycin and 1% L-glutamine. The endometrial cancer cell line (Ishikawa) was cultured in minimum essential medium (MEM) supplemented with 5% FBS, 1% Non-Essential Amino Acids (NEAA), 500 units/ml penicillin and 500 μg/ml streptomycin. Cells were grown at 37°C in a humidified 95% air/5% CO_2_ incubator.

### Cell treatments

Ovarian cancer cell lines (OVCAR-8 and IGROV-1) and an endometrial cancer cell line (Ishikawa) at 65% confluence were treated with the global genomic DNA demethylating agent, 5-aza-2‵-deoxycytidine (DAC) (Sigma-Aldrich, St. Louis, MO, USA), or the histone deacetylase inhibitor, Trichostatin A (TSA). For 5-aza-2‵-deoxycytidine treatment, cells were seeded and incubated overnight in growth media. The cells were then left untreated or treated with 5 μmol/L of DAC for 24 hours on day 2. The culture was re-dosed every 48 hours (days 4 and 6) and the medium was changed 24 hours after adding DAC. Cells were harvested on day 8 for RNA isolation. For TSA treatment, cells were seeded and incubated in their respective growth media containing TSA (500 ng/ml) for 24 hours. At the conclusion of either treatment, cells were harvested for the analysis of RNA, genomic DNA, and protein as described below.

### Methylation-specific polymerase chain reaction

**Figure 1:**
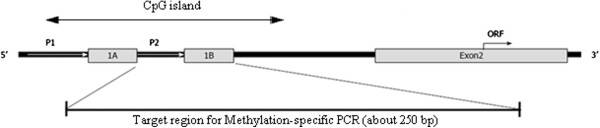
**Schemes of the CD133 promoter region (modified from Pleshkan et al.) [**[12]**]**.

For methylation analysis, 2 μg of genomic DNA obtained from gynecologic cancer cell lines were modified using the EZ DNA Methylation™ Kit (Zymo Research, Orange, CA, USA). Primers specific for bisulfite modified DNA were designed using MethPrimer software (http://www.urogene.org/methprimer/index1.html). The primers used in this study (located −8061 to −7782 from transcription start site, modified from Pleshkan et al. [12]) were as follows: CD133 M primer sense (5^′^-TTCGGGATAGAGGA AGTCGTAA-3^′^) and CD133 M primer antisense (5^′^-CTCCCGCCCTAATCACCGCT-3^′^); and CD133 U primer sense (5^′^-TTTGGGATAGAGGAAGTTGTAA-3^′^) and CD133 U primer antisense (5'-CTCCCACCCTAATCACCACT-3') (Figure 1). The PCR conditions were as follows: 94°C for 5 minutes, followed by 43 cycles of 94°C for 30 seconds, 60°C (methylation-specific PCR, MSP) or 62°C (unmethylation-specific PCR, USP) for 30 seconds, and 72°C for 60 seconds, and finally 72°C for 7 minutes. The amplified DNA fragments were fractionated on 2% agarose gels and stained with ethidium bromide. 

### Flow cytometry (FACS) analysis

A total of 1 × 10^6^ cells were incubated with phosphate buffered saline (PBS) containing 1% bovine serum albumin (BSA) alone or with mouse antihuman CD133/2-PE (Miltenyi Biotech, Auburn, CA, USA) for 45 minutes at 4°C. Cells were washed extensively (three times in PBS) and incubated with the appropriate PE-conjugated second antibody for 45 minutes at 4°C in the dark. Control cells were incubated with immunoglobulin G (IgG). After washing the cells extensively, they were analyzed on a FACScan instrument (Becton, Dickinson and Company, Mountain View, CA, USA). Data from 10,000 cells was collected for each sample.

### Total RNA isolation and reverse-transcriptase reaction

RNA was extracted and purified using an RNeasy mini kit (Qiagen, Valencia, CA, USA) as described in the manufacturer’s protocol. The concentration of RNA was measured using a spectrophotometer (DU®530; Beckman, Fullerton, CA, USA). The total RNA sample (1 μg/sample) was used to generate cDNA with the SuperScript^™^ III First-Strand Synthesis System for RT-PCR kit (Invitrogen, Carlsbad, CA, USA). Briefly, RNA was reverse-transcribed in a mixture of 25 mM MgCl_2_, 10 mM dNTP mix, 10 × RT buffer, 0.1 M DTT, 200 U of Super Script^™^ III (Invitrogen, Carlsbad, CA, USA), 40 U of RNase Out, 50 μL Moligod (T) primers at a final volume of 20 μL. The reaction was run at 65°C for 5 minutes and 50°C for 50 minutes, and then the enzyme was heat inactivated at 85°C for 5 minutes. Four microliters of each reaction product was used for real-time PCR.

### Reverse transcription-polymerase chain reaction

For CD133 expression analysis, cDNA was amplified in a 25 μL PCR reaction containing 2 μL of the reverse-transcription reaction mix, primers and 1 unit of *Taq* DNA polymerase. Reverse transcription-polymerase chain reaction (RT-PCR) was carried out using RT-specific primers, CD133 RT sense (5^′^-CTGGGGCTGCTGTTTATTA-3') and CD133 RT antisense (5^′^-TACCTGGTGATTTGCCACAA-3'). PCR conditions consisted of 5 minutes at 95°C for initial denaturation, followed by 35 cycles of 95°C (30 seconds), 54°C (30 seconds), and 72°C (30 seconds) and a final elongation of 4 minutes at 72°C. PCR amplification was performed in a programmable thermal cycler (PCR System 9700; Applied Biosystems; Foster City, CA, USA). Primers for GAPDH were used to confirm RNA integrity. Both CD133 and GAPDH RT-PCR reactions were performed using the same cDNA synthesis reactions. Amplified DNA fragments were fractionated on 2% agarose gels and stained with ethidium bromide.

### Quantitative real-time PCR analysis

Quantitative real-time PCR was used to quantify CD133 expression. CD133 expression was normalized using the GAPDH housekeeping gene product as an endogenous reference. The primers and probes were designed for human CD133 using Primer Express 2.0 (Applied Biosystems, Foster City, CA, USA). CD133 mRNA levels were quantified using TaqMan Real-Time PCR with an ABI 7300 system instrument (Applied Biosystems). Gene-specific probes and primer pairs for CD133 (Assays-on-Demand, Hs01009250_m1; Applied Biosystems) were used. For each probe/primer set, a standard curve was generated, which confirmed the linear increase in amplification with increasing amounts of cDNA. The amplification conditions were 2 minutes at 50°C, 10 minutes at 95°C, and a two-step cycle of 95°C for 15 seconds and 60°C for 60 seconds for a total of 45 cycles.

### Western blot analysis

Total cell lysates were prepared by sonication. Briefly, cells were lysed in buffer containing 50 mM HEPES (pH 7.5), 150 mM NaCl, 1.5 mM MgCl_2_, 1 mM EDTA, 10% glycerol, 1% Triton X-100, and a mixture of protease inhibitors (aprotinin, PMSF, and sodium orthovanadate). The protein concentrations of the resulting cell lysates were measured by the Bradford assay. Equal amounts of total protein were resolved on a 10% SDS-polyacrylamide gel. Next, proteins were transferred to nitrocellulose membranes (Hybond^™^-P; Amersham Biosciences, Piscataway, NJ, USA). After blocking (TBS, 0.1% Tween 20) at 4°C for 1 hour, the membranes were incubated with anti-human CD133 (dilution 1:1000) and β-actin (dilution 1:3000) primary antibodies. After incubation, the blots were washed (TBS, 0.1% Tween 20) and incubated with secondary antibodies linked to HRP (dilution 1:2000; Bio-Rad Laboratories, Hercules, CA, USA). The blots were exposed to X-ray film for visualization.

## Results

**Figure 2:**
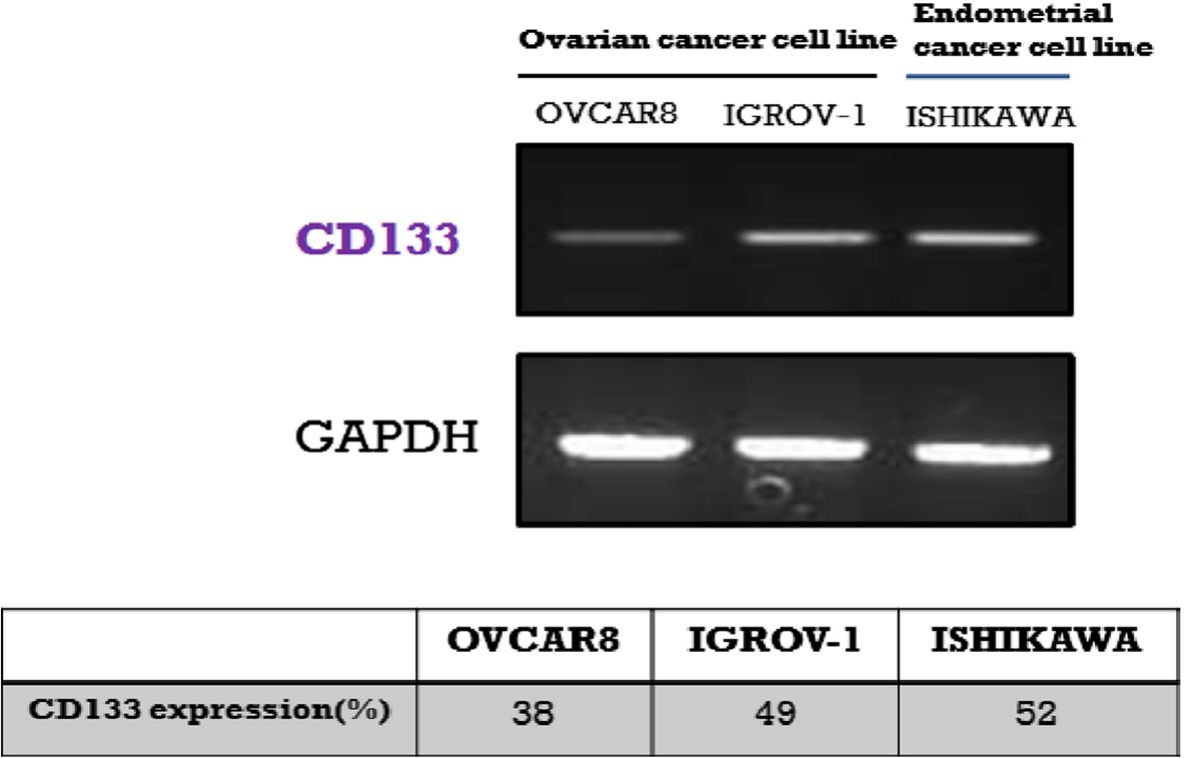
Expression analysis of CD133 in ovarian (OVCAR-8 and IGROV-1) and endometrial (Ishikawa) cancer cell lines.

We analyzed the expression of CD133 in three gynecologic cancer cell lines by RT-PCR, quantitative real-time PCR, western blot, and FACS analysis. CD133 expression was examined in ovarian cancer cell lines (OVCAR-8 and IGROV-1) and Ishikawa cells and normalized to GAPDH expression. Although each of these cell lines is of an adenocarcinoma origin, the CD133 mRNA expression varied significantly among the cell lines, with the weakest expression observed in OVCAR-8 cells and the strongest expression in Ishikawa cells (Figure 2).

**Figure 3:**
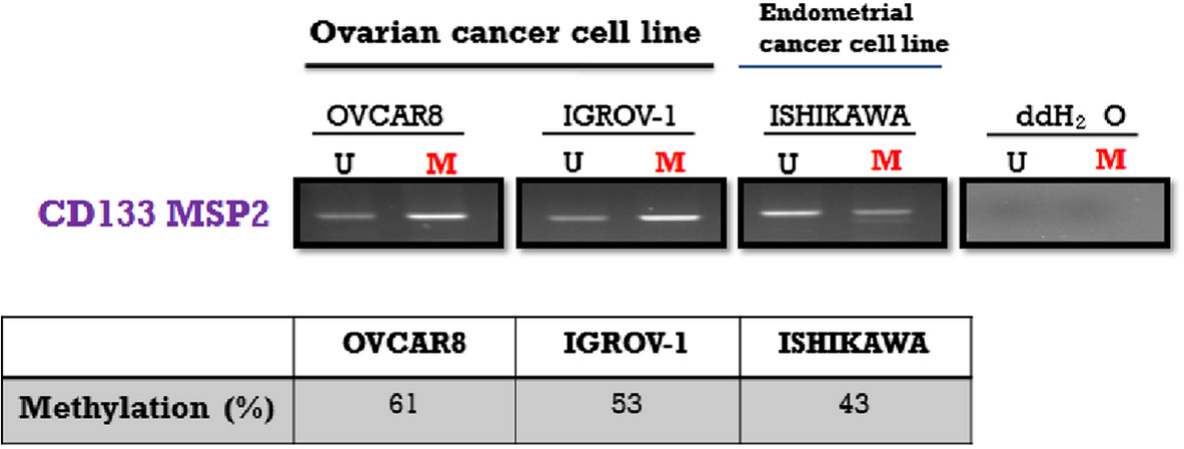
CD133 promoter methylation-specific PCR (MSP) in ovarian (OVCAR-8 and IGROV-1) and endometrial (Ishikawa) cancer cell lines.

To investigate the correlation of CD133 expression with CD133 promoter methylation, methylation-specific PCR was conducted on the CD133 P2 promoter. The CD133 P2 promoter used in this study had a size of approximately 250 bp, and its approximate location and size are depicted in Figure 1. The degree of methylation of the CD133 P2 promoter was observed to be 61% in OVCAR-8 cells, 53% in IGROV-1 cells, and 43% in Ishikawa cells. Thus, while not statistically significant, higher levels of methylation were observed in the ovarian cancer cell lines compared with the endometrial cancer cell line (Figure 3).

**Figure 4:**
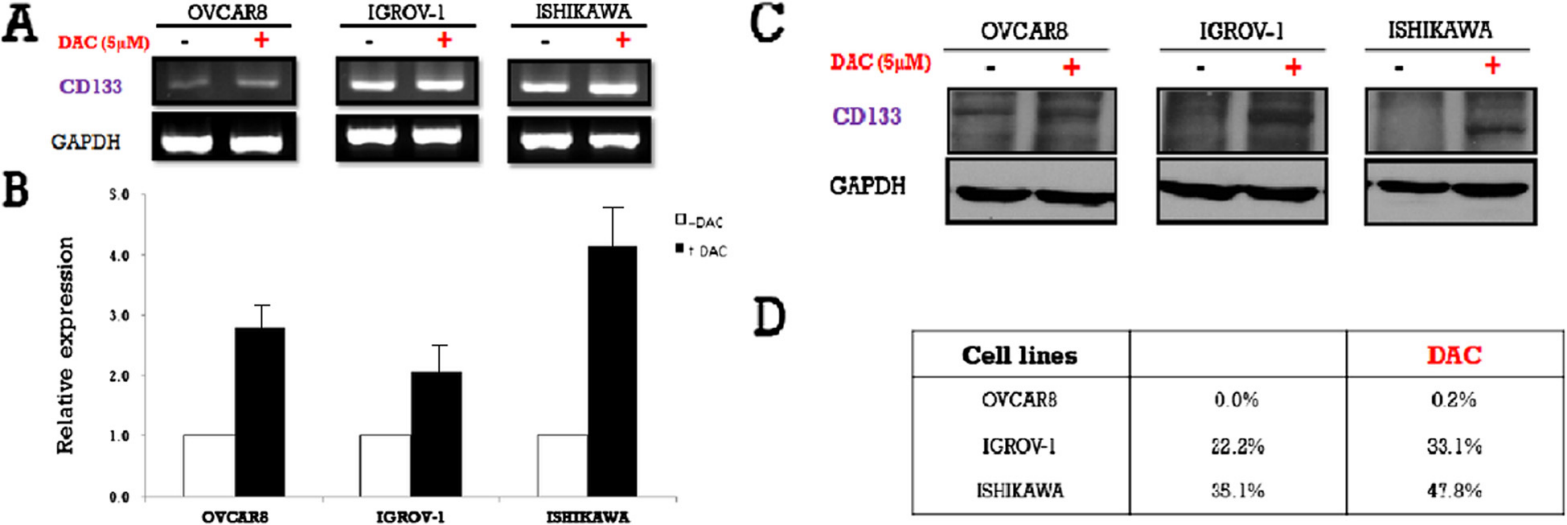
**CD133 mRNA and protein expression in ovarian (OVCAR-8 and IGROV-1) and endometrial (Ishikawa) cancer cell lines before and after treatment with 5-aza-2`-deoxycytidine (DAC).A**: Reverse transcription-polymerase chain reaction (RT-PCR) analysis of the CD133 gene in three cell lines with or without 5-aza-2‵-deoxycytidine (DAC). **B**: Representative results of real-time PCR analysis of CD133 expression in three cell lines with or without 5-aza-2‵-deoxycytidine (DAC). **C**: Immunoblot analysis of CD133 expression in three cell lines with or without 5-aza-2‵-deoxycytidine (DAC). **D**: Fluorescence-activated cell sorting analysis of CD133+ cells in three cell lines with or without 5-aza-2‵-deoxycytidine (DAC). * Results are representative of three independent experiments.

**Figure 5:**
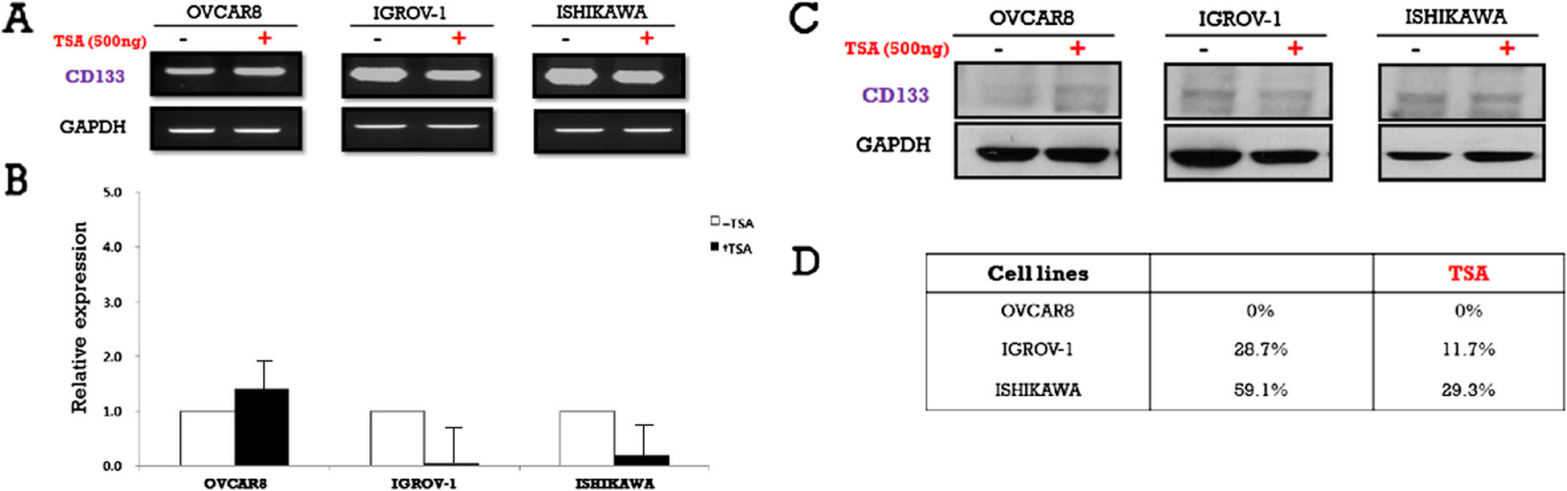
**CD133 mRNA and protein expression in ovarian (OVCAR-8 and IGROV-1) and endometrial (Ishikawa) cancer cell lines before and after treatment with Trichostatin A (TSA).A**: Reverse transcription-polymerase chain reaction (RT-PCR) analysis of the CD133 gene in three cell lines with or without Trichostatin A (TSA). **B**: Representative results of real-time PCR analysis of CD133 expression in three cell lines with or without Trichostatin A (TSA). **C**: Immunoblot analysis of CD133 expression in three cell lines with or without Trichostatin A (TSA). **D**: Fluorescence-activated cell sorting analysis of CD133+ cells in three cell lines with or without Trichostatin A (TSA). * Results are representative of three independent experiments.

**Figure 6:**
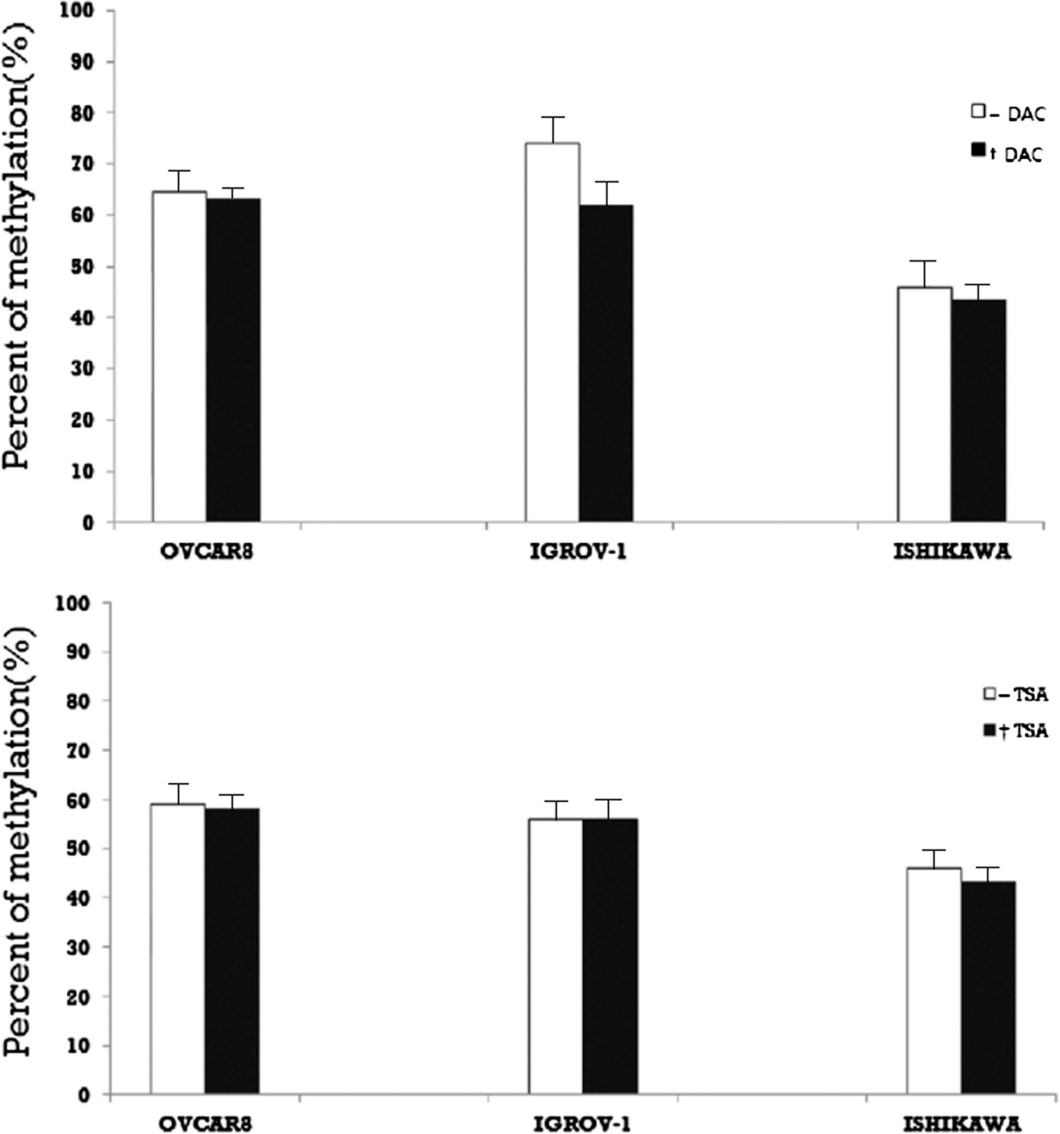
**Methylation of the CD133 P2 promoter in ovarian (OVCAR-8 and IGROV-1) and endometrial (Ishikawa) cancer cell lines before and after treatment with 5-aza2‵-deoxycytidine (DAC) and Trichostatin A (TSA).** * Representative results of real-time PCR analysis revealing CD133 promoter methylation in three cell lines with or without 5-aza-2`-deoxycytidine (DAC) and Trichostatin A (TSA). * Results are representative of three independent experiments.

To investigate the potential epigenetic regulation of CD133, its expression was examined in the three cell lines following treatment with either DAC or TSA. CD133 expression after DAC treatment was increased on both the mRNA and protein levels. On the contrary, CD133 mRNA expression was decreased after TSA treatment in all cell lines except OVCAR-8. However, there was no change in CD133 protein expression following TSA treatment in OVCAR-8 cells, while the other two cell lines exhibited decreased CD133 expression. (Figures 4 and 5). In addition, MSP of the CD133 P2 promoter revealed that methylation was reduced after treatment with either drug (Figure 6).

**Figure 7:**
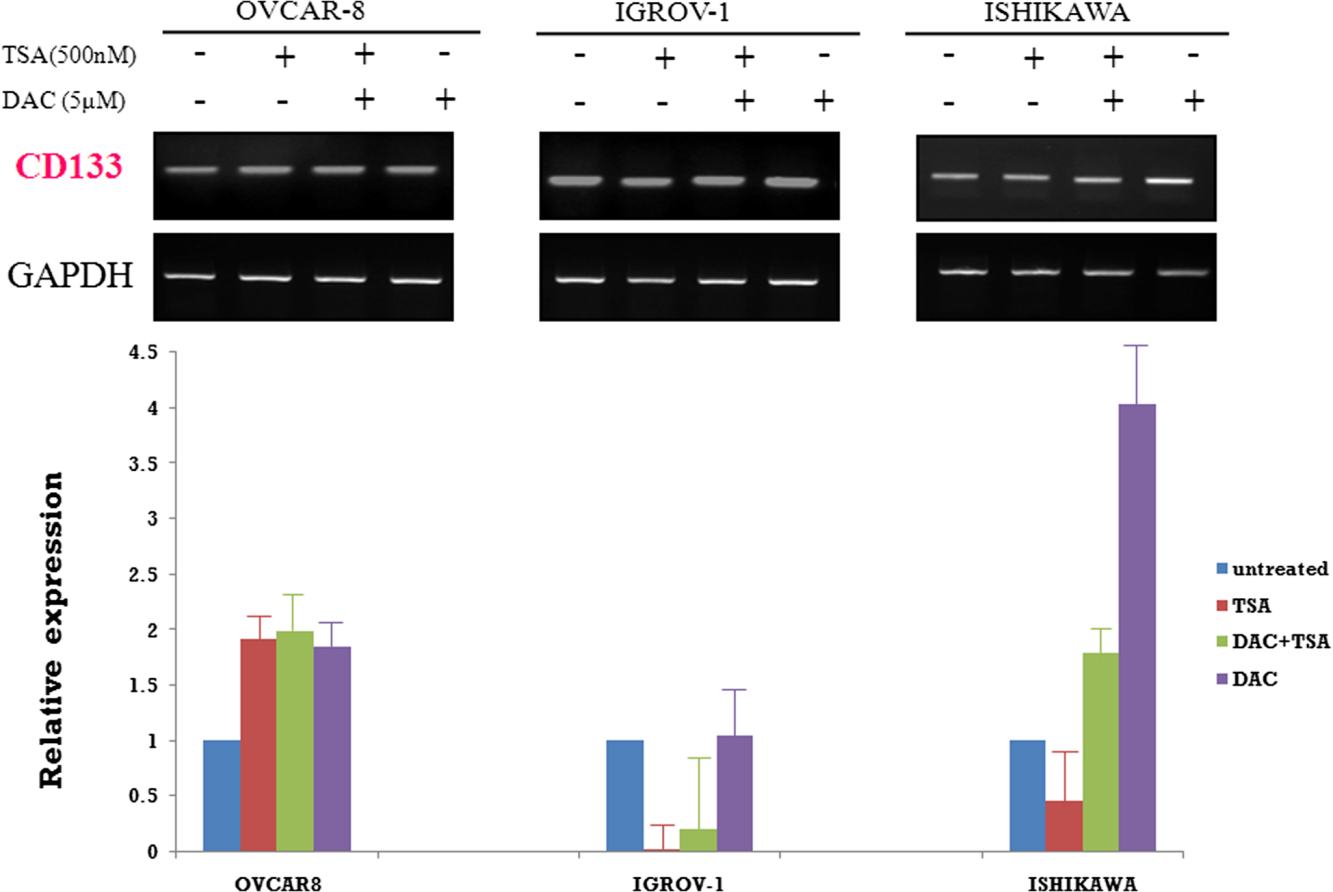
**CD133 mRNA expression in ovarian (OVCAR-8 and IGROV-1) and endometrial (Ishikawa) cancer cell lines after treatment with Trichostatin A (TSA), 5-aza-2`-deoxycytidine (DAC), or both.** * Representative results of real-time PCR analysis of CD133 expression in three cell lines with or without 5-aza-2`-deoxycytidine (DAC) and Trichostatin A (TSA). * Results are representative of three independent experiments.

Treatment with DAC led to an increase in cell surface expression of CD133 in all three cancer cell lines, but treatment with TSA led to such an increase only in OVCAR-8 cells. Combined drug treatment was synergistic for increasing cell surface CD133 expression in OVCAR-8. In IGROV-1 and Ishikawa cells, the effect of DAC was found to be greater than that of TSA (Figure 7).

## Discussion

This is a unique study analyzing the expression of CD133 antigen in primary ovarian and endometrial cancer cell lines and its potential regulation by epigenetics. Interestingly, treatment with 5-aza-2‵-deoxycytidine (DAC) increased CD133 expression, while Trichostatin A (TSA) treatment resulted in variable regulation of CD133. In addition, the expression of CD133 was negatively correlated with the degree of methylation on the CD133 P2 primer.

Cancer stem cells (CSCs) are cancer cells that possess characteristics associated with normal stem cells, specifically the ability to give rise to all cell types found in a particular cancer sample [13]. CSCs are therefore tumorigenic, perhaps in contrast to other non-tumorigenic cancer cells. CSCs may generate tumors through the stem cell processes of self-renewal and differentiation into multiple cell types. Such cells are proposed to persist in tumors as a distinct population and cause relapse and metastasis by giving rise to new tumors. Therefore, development of specific therapies targeted at CSCs holds hope for improving the effects of cancer treatment, especially for recurrent and metastatic disease [3].

CD133, a cell surface antigen, is a glycoprotein also known in humans and rodents as prominin 1 (PROM1), and was first isolated from hematopoietic stem cells [14]. The function of CD133 is currently being evaluated in several cancers. It is regarded as a CSC marker in colorectal carcinoma and glioblastoma [15,16]. Given the strong rationale linking CD133 expression to more aggressive cellular behavior, including resistance to chemotherapy and radiotherapy, a direct correlation between CD133 expression and advanced disease stage as well as poor differentiation grade has been shown in hepatocellular carcinoma [17]. Further, several studies detected the expression of CD133 in ovarian cancer cells of different origins [18-20], suggesting that CD133 expression in ovarian cancer is directly regulated by epigenetic modifications and that CD133 is a candidate marker of ovarian cancer stem cells. Shmelkov et al. characterized CD133 as having five alternative promoters (P1–P5) that are active in a tissue-dependent manner [21]. As Shmelkov’s result, because CD133 P2 promoter is the only active promoter in ovarian tissues, we investigated the effect of promoter methylation in P2 region.

Based on the above findings, we hypothesized that CD133 is regulated through P2 promoter methylation by epigenetic modification in ovarian cancer. Thus, we selected several ovarian cancer cell lines in which to evaluate CD133 expression. Following treatment of cells with DAC, increased expression of CD133 was observed, similar to previous studies [6,15,20]. However, the TSA had a variable effect on CD133 expression that varied by cell line. These results suggest that TSA may have different roles in cells depending on cell type. One possible mechanism explaining this differential effect is that TSA inhibits the proliferation of tumor cells in culture and in vivo by inducing cell cycle arrest, differentiation, and/or apoptosis. Another potential mechanism is that TSA may not only modify histone acetylation, but also alter DNA methylation in tumorigenic cells, but not in normal cells. Thus, TSA may promote the expression of apoptosis-related genes, leading to cancerous cells surviving at lower rates, ultimately slowing cancer progression [22]. Finally, there is the possibility that experimental error occurred. We conducted experiments in triplicate using different concentrations of TSA, treatment times, and incubation times to rule out the possibility of error, and similar results were obtained under all of the conditions tested. Thus, further experiments will be required to elucidate the exact mechanism underlying the differential effect of TSA treatment in different cancer cell lines, including sorting cells according to CD133 expression.

## Conclusions

In conclusion, the expression of CD133 antigen in primary ovarian and endometrial cancer cell lines is regulated by epigenetics. Specifically, CD133 expression was increased by 5-aza-2`-deoxycytidine (DAC), but inversely regulated in a cell-line dependent manner by Trichostatin A (TSA). In addition, the expression of CD133 was negatively correlated with the degree of methylation of the CD133 P2 promoter.

## Competing interests

The authors declare that they have no competing interests.

## Authors' contributions

KJM: AB, JY, and ES. KAS: AB, JY, and ES. Yung-Taek Ouh: AB, JY, and ES. JHH: AB, JY, and ES. JKL: AB, JY, ES, and FG. All authors read and approved the final manuscript.
